# Increased TOX expression associates with exhausted T cells in patients with multiple myeloma

**DOI:** 10.1186/s40164-022-00267-0

**Published:** 2022-03-04

**Authors:** Yujie Zhao, Pengjun Liao, Shuxin Huang, Tairan Deng, Jiaxiong Tan, Youxue Huang, Huien Zhan, Yangqiu Li, Shaohua Chen, Liye Zhong

**Affiliations:** 1 Key Laboratory for Regenerative Medicine of Ministry of Education, School of Medicine, Institute of Hematology, Jinan University, Guangzhou, 510632 China; 2 Department of Hematology, Guangdong Academy of Medical Sciences, Guangdong Provincial People’s Hospital, Guangzhou, 510080 China; 3 Department of Hematology, First Affiliated Hospital, Jinan University, Guangzhou, 510632 China

**Keywords:** TOX, Multiple myeloma, PD-1, Tim-3, CD244, T cell exhaustion

## Abstract

Previous studies have shown increased aberrant expression of immune checkpoint (IC) proteins, such as programmed cell death receptor-1 (PD-1) and T cell immunoglobulin mucin-domain-containing-3 (Tim-3) on T cells from patients with multiple myeloma (MM), which result in T cell exhaustion and dysfunction. However, little is known about the mechanism regulating aberrant IC protein expression. In this study, we analyzed the expression of TOX (thymocyte selection-associated HMG BOX), a crucial transcription factor involved in T cell exhaustion, and its co-expression with PD-1, Tim-3, and CD244 in T cell subsets by multi-color fluorescent flow cytometry in peripheral blood (PB) and bone marrow (BM) samples from patients with MM. Significantly, the percentage of TOX + CD3 +/CD4 +/CD8 + T cells was increased, and similarly, higher numbers of TOX co-expression with PD-1, Tim-3, and CD244 on CD3 +/CD4 +/CD8 + T cells were found. Interestingly, the numbers of TOX +, TOX + PD-1 +, and TOX + Tim-3 + regulatory T (Treg) cells also significantly increased in both the PB and BM of MM patients. In summary, we for the first time observed increased TOX expression concurrent with PD-1, Tim-3, and CD244 on T cells, which may contribute to T cell exhaustion and impair their function in MM. Thus, TOX may be considered a potential target for reversing T cell exhaustion and improving T cell function in MM.

**Supplementary Information:**

The online version contains supplementary material available at 10.1186/s40164-022-00267-0.

## To the Editor,

**Fig. 1:**
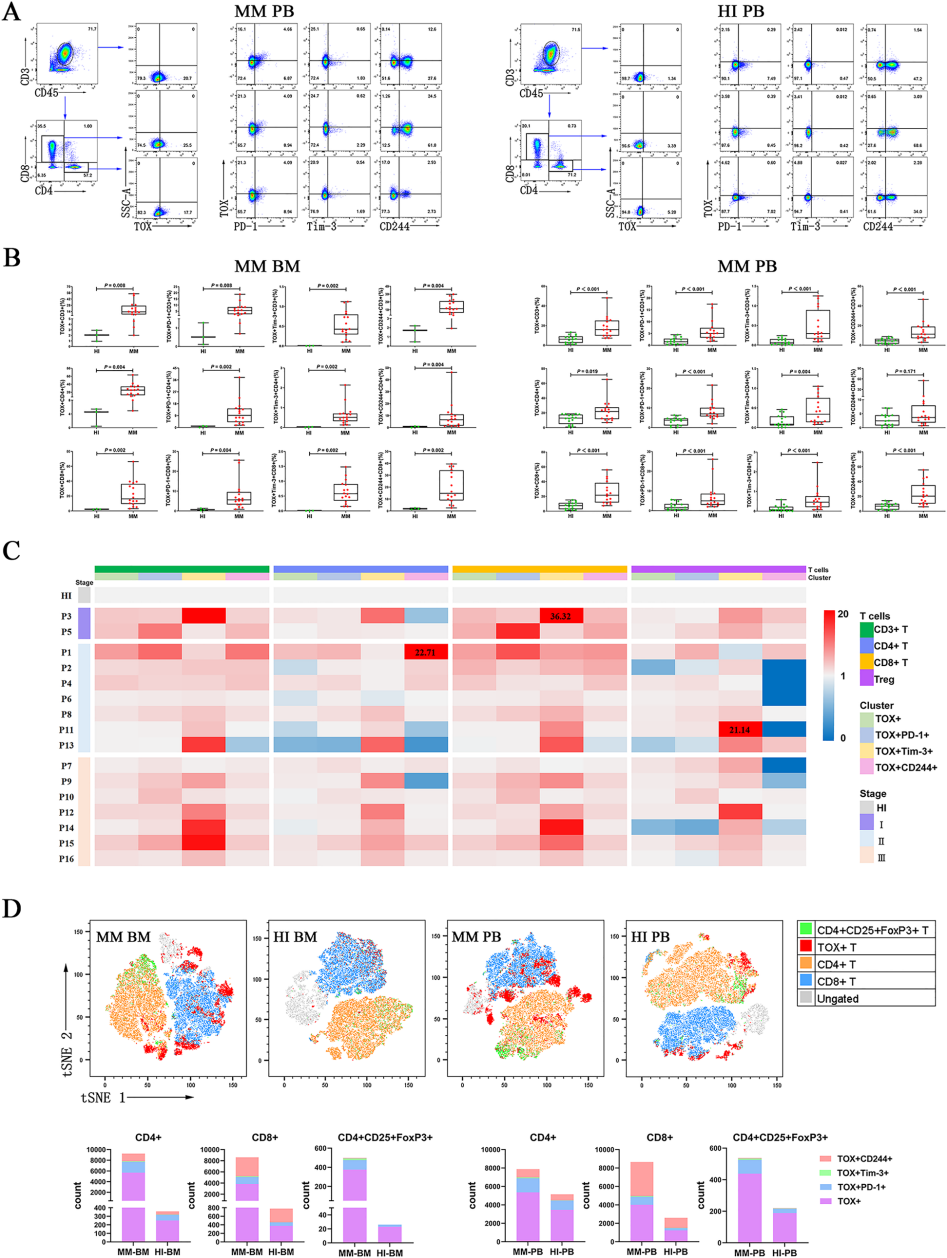
Distribution and frequency of TOX expression and co-expression with PD-1, Tim-3, and CD244 in T cell subsets in PB and BM from patients with MM. **A** The analytic logic of flow cytometry detection of TOX expression and co-expression with PD-1, Tim-3, and CD244 in CD3 +, CD4 +, and CD8 + T cell subsets in PB from a patient with MM and a healthy individual (HI). **B** Comparison of the percentage of TOX + CD3 + T cells (median: BM: 13.2 vs 2.06, *P* = 0.008, PB: 15.9 vs 6.145, *P* < 0.001), TOX + CD4 + T cells(median: BM: 25.2 vs 4.57, *P* = 0.004, PB: 21.85 vs 12.95, *P* = 0.019), TOX + CD8 + T cells (median: BM: 16.2 vs 2.23, *P* = 0.002, PB: 21.6 vs 7.51, *P* < 0.001), TOX + PD-1 + CD3 + /CD4 + /CD8 + T cells (median: BM: 5.575/9.19/5.6 vs 0.51/1.02/0.6, *P* = 0.008, *P* = 0.002, *P* = 0.004, respectively, PB: 5.04/7/4.985/ vs 1.495/3.76/1.555, *P* < 0.001, *P* < 0.001, *P* < 0.001, respectively), TOX + Tim-3 + CD3 + /CD4 + /CD8 + T cells (median: BM: 0.425/0.51/0.58 vs 0.00706/0.03/0.00747, *P* = 0.002, *P* = 0.002, *P* = 0.002, respectively, PB: 0.295/0.34/0.455 vs 0.063/0.0865/0.068, *P* < 0.001, *P* = 0.004, *P* < 0.001, respectively), and TOX + CD244 + CD3 + /CD4 + /CD8 + T cells (median: BM: 11.35/6.535/14.8 vs 1.71/0.81/1.85, *P* = 0.004, *P* = 0.004, *P* = 0.002, respectively, PB: 11.2/3.645/20.25 vs 4.36/2.435/6.755, *P* < 0.001, *P* = 0.171, *P* < 0.001, respectively) in BM and PB from patients with MM and HIs. **C** Heatmap representing the frequency of TOX +, TOX + PD-1 +, TOX + Tim-3 +, and TOX + CD244 + cells in T cell subsets in PB from 16 patients (stage I (2 cases), stage II (7 cases) and stage III (7 cases) with MM compared with HIs. **D** tSNE clusters of the global distribution and frequency of different phenotypes of T cells in the BM and PB of patients with MM and HIs. Note: P1–P16: MM patients who are numbered according to collection time

**Fig. 2:**
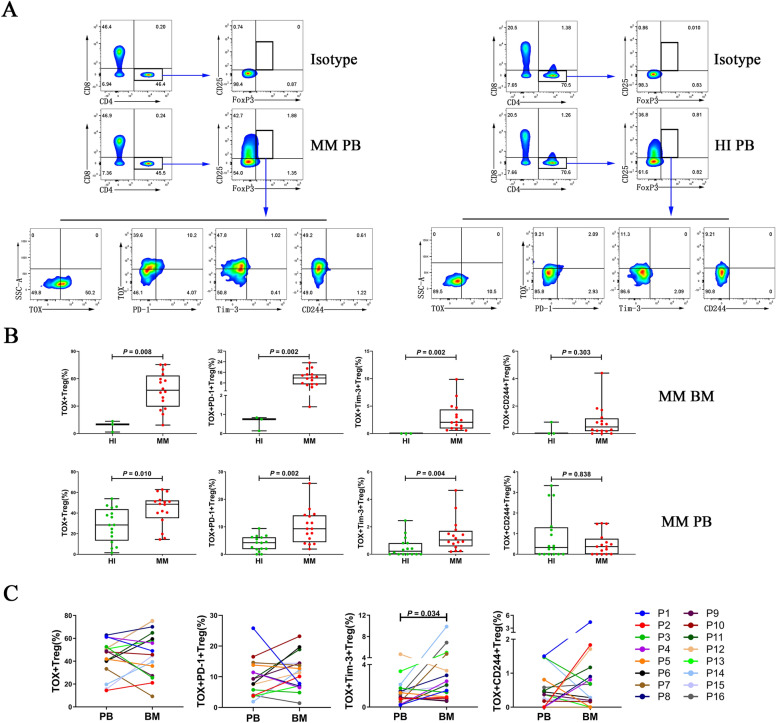
Distribution and frequency of TOX expression and co-expression with PD-1, Tim-3, and CD244 on Treg cells in BM and PB from MM patients. **A** The analytic logic of flow cytometry detection of TOX expression and co-expression with PD-1, Tim-3, and CD244 in Treg cells in PB from a patient with MM and a HI. **B** Comparison of the percentage of TOX + Treg cells (median: BM: 47.35 vs 10, *P* = 0.008, PB: 48.55 vs 28.45, *P* = 0.010), TOX + PD-1 + Treg cells (median: BM: 11.75 vs 0.75, *P* = 0.002, PB: 9.3 vs 4.21, *P* = 0.002), TOX + Tim-3 + Treg cells (median: BM: 2.045 vs 0, *P* = 0.002, PB: 1.04 vs 0.22, *P* = 0.004), and TOX + CD244 + Treg cells (median: BM: 0.48 vs 0, *P* = 0.303, PB: 0.37 vs 0.325, *P* = 0.838) in BM and PB from patients with MM and HIs. **C** Comparison of the percentage of TOX +, TOX + PD-1 +, TOX + Tim-3 +, and TOX + CD244 + Treg cells between PB and BM from 16 patients (P1–P16) with MM

Multiple myeloma is an aggressive, malignant, and incurable disease characterized by neoplastic plasma cell clone proliferation [1]. Poor prognoses of MM patients may be related to T cell immunodeficiency [2]. Recent findings have indicated that aberrant expression of immune checkpoint (IC) proteins such as programmed cell death receptor-1 (PD-1) and T cell immunoglobulin mucin-domain-containing-3 (Tim-3) is a key reason for T cell immune suppression though the promotion of T cell exhaustion [2, 3]. Up-regulation of PD-1 and other IC proteins, such as Tim-3, on CD4 + and CD8 + T cells has been detected in PB from patients with MM [2, 4]. Immunotherapy based on targeting ICs, such as PD-1 blockade, improves the clinical outcome of solid tumors and lymphoma in clinical trials, and the underlying mechanism is thought to reverse the immunosuppressive status of T cells and restore their anti-tumor ability in patients [5]. However, even with PD-1 over-expression on exhausted T cells, the effects of PD-1 blockade appear to be limited and heterogeneous for MM patients [6, 7]. These observations may be related to different immunosuppressive microenvironments and the expression pattern of ICs between solid tumors and MM [2]. Recently, it has been reported that over-expression of TOX (thymocyte selection-associated HMG BOX), a crucial transcription factor involved in T cell exhaustion, is detected in CD8 + tumor-infiltrating lymphocytes (TILs) in bladder cancer, and this is related to PD-1 expression on T cells [8, 9]. To further characterize the alternative expression profile of IC proteins and co-expression with their regulatory factors, we analyzed the expression of TOX and TOX co-expression with PD-1, Tim-3, and CD244 in T cells by multi-color fluorescent flow cytometry in peripheral blood (PB) and bone marrow (BM) samples from 16 patients with MM (Additional file 1: Supplementary Methods and Additional file 3: Table S1). Significantly, the percentages of TOX + CD3 +/CD4 +/CD8 + T cell subsets were all increased, and higher numbers of TOX co-expressed with PD-1, Tim-3, or CD244 in CD3 +/CD4 +/CD8 + T cells were found in both PB and BM from patients with MM in comparison with healthy controls (Fig. 1A, B). This result is consistent with the finding of up-regulation of TOX in solid tumors and lymphomas [10]. However, as the heatmap shows in Fig. 1C, the frequency of TOX and co-expression with PD-1, Tim-3, and CD244 in CD3 +, CD4 +, and CD8 + T cells relatively varied between different MM patients, and did not appear to be associated with different stages of MM. Interestingly, a higher frequency of the TOX + T cell subset can be also found in stage I MM (Fig. 1C). The global distribution and frequency of different phenotypes of T cells in the BM and PB of patients with MM and HI can be represented by tSNE clusters (Fig. 1D). Our previous study demonstrated that the level of PD-1 + Tim-3 + CD3 +/CD4 +/CD8 + T cells was high in the BM when compared with PB [2]. In this study, we also compared the percentage of the TOX + T cell subsets in 16 pairs of PB and BM samples from MM patients. Overall, a high percentage of TOX + T cell subsets could be found in the BM in comparison with that in PB in most cases; however, these were not statistically significant (Fig. 1B, Additional file 2: Figure S1) except for TOX + Tim-3 + regulatory T (Treg) cells, which were significantly higher in BM than in PB (Fig. 2C). Interestingly, the numbers of TOX + Tregs and TOX + PD-1 +/Tim-3 + Tregs significantly increased in the PB and BM (Fig. 2A, B). Our previous study also revealed an increase in TOX + Treg cells in patients with lymphoma [11]. However, the role of Treg cells with higher TOX and PD-1 or Tim-3 in MM pathogenesis, prognosis, and treatment remains unclear. Unlike high expression of PD-1 on CD8 + T cells induces exhaustion, PD-1 expression on Tregs negatively impacts immunosuppressive functions [11], moreover, TOX promotes the exhaustion of antitumor CD8 + T cells by preventing PD-1 degradation due to the binding of TOX to PD-1 in the cytoplasm and maintaining abundant PD-1 expression at the T cell surface [12]. Suggesting that the role of TOX may contribute to maintain PD-1 expression on Treg cells which may enhance negative immune regulatory in MM. In this case, whether targeting TOX has a dual inhibitory function remains an open question.

Taken together, first, our findings indicate increased TOX expression in T cells in MM patients. Second, TOX co-expression with PD-1, Tim-3, and CD244 in T cells may be involved in promoting T cell exhaustion and impairing their function in MM. Third, higher TOX + Treg subsets in the BM, may contribute to mediating the BM immunosuppressive microenvironment, which may also be a reason why the effects of PD-1 blockade are relatively different in different MM patients. Understanding the exhausted phenotype pattern of T cells in different MM patients may help guide precision immunotherapy for MM patients.

In summary, we characterized the distribution of TOX expression in T cell subsets in MM patients. Increased TOX concurrent with PD-1, Tim-3, and CD244 in T cells may be considered a potential target for reversing T cell exhaustion and improving T cell function in MM.

## Supplementary Information

**Additional file 1: Supplementary methods.**

**Additional file 2: Figure S1.** Comparison of the percentage of the TOX +, TOX + PD-1 +, TOX + Tim-3 +, and TOX + CD244 + CD3 +/CD4 +/CD8 + T cell subsets between PB and BM from 16 patients (P1–P16) with MM.

**Additional file 3: Table S1.** Clinical information of MM patients used in the study.

## Data Availability

The materials supporting the conclusions of this research article are included within the article.

## Abbreviations

BM: Bone marrow
IC: Immune checkpoint
MM: Multiple myeloma
PB: Peripheral blood
PD-1: Programmed cell death receptor-1
TILs: Tumor-infiltrating lymphocytes
Tim-3: T cell immunoglobulin mucin-domain-containing-3
TOX: Thymocyte selection-associated HMG BOX
Treg: Regulatory T
