# Supplementary material for: Increased TOX expression associates with exhausted T cells in patients with multiple myeloma
Source: Exp Hematol Oncol. 2022 Mar 4;11:12. doi: 10.1186/s40164-022-00267-0 (PMC8895562; doi:10.1186/s40164-022-00267-0)
Supplement: Supplementary file 1 — Additional file 1: Supplementary methods. [file 40164_2022_267_MOESM1_ESM.doc]

**Supplementary**

**METHODS**

1 Samples

Peripheral blood (PB) and bone marrow (BM) samples were collected from 16 newly diagnosed, untreated MM patients including 10 males and 6 females (median age: 62 years, range: 36-73 years) named P1 to P16. The clinical information of the MM patients is listed in Supplementary Table S1. PB samples from 16 healthy individuals (HIs), including 9 males and 6 females (median age: 57 years, range: 31-82 years) served as PB controls. BM samples from 2 non-tumor surgery cases and 1 healthy donor, including 1 male and 2 females (median age: 68 years, range: 27-76 years), served as BM controls. All PB and BM samples were obtained with informed consent, and ethical approval was obtained from the Ethics Committee of Guangdong Provincial Pepple’s Hospital, Guangzhou, China.

2 Antibodies

The antibodies used for this study, including CD45-BV605 (clone HI30), CD3-APC-Cy7 (clone SK7), CD8-APC-R700 (clone RPA-T8), CD25-BB515 (clone 2A3), PD-1-PE-Cy7 (clone EH12.1), FoxP3-BB700 (clone 236A/E7), BB515 isotype control (clone X40), PE-Cy7 isotype control (clone MOPC-21), and the BB700 isotype control (clone X40), were purchased from BD Biosciences (San Jose, CA). CD4-BV510 (clone SK3), CD244-PE (clone C1.7), Tim-3-BV421 (clone F38-2E2), and the BV421 isotype control (clone MOPC-21) were purchased from BioLegend (San Diego, CA). TOX-eFluor 660 (clone TXRX10) and the eFluor 660 isotype control (clone eBR2a) were purchased from eBioscience (San Diego, CA).

3 Immunofluorescence staining for flow cytometry

Immunofluorescence staining was performed according to the manufacturer’s instructions. Briefly, a pre-mixed solution combining cell surface antibody and staining buffer (BD Biosciences, San Jose, CA) with 100 μL of blood samples from patients with multiple myeloma was used, and it was incubated at room temperature for 15 min in the dark. Next, 2 mL 1× red blood cell (RBC) lysis buffer (BD Biosciences, San Jose, CA) was added to resuspend the stained sample, which was then incubated at room temperature for 10 min and then washed once with 2 mL 1×PBS and centrifuged. The cells were then fixed and permeabilized by 1x Fix/Perm Buffer (BD Biosciences, San Jose, CA) for 50 minutes at 4°C in the dark, and they were then washed with 1x Perm/Wash Buffer. Finally, intranuclear staining of TOX and FoxP3 was performed for 50 minutes at 4°C in the dark followed by washing twice with 1X Perm/Wash Buffer (BD Biosciences, San Jose, CA) and resuspension in 0.3 mL 1X PBS (GenXionCell, Guangzhou, China) to prepare for flow cytometry analysis. A total of 50,000 CD45+CD3+ cells were acquired with a BD FACS Canto flow cytometer (BD Biosciences, San Jose, CA) and analyzed by Flowjo software (Flowjo LLC, Ashland, OR). Before obtaining the target CD45+CD3+ T cells, dead and sticky cells were eliminated by FSC-A/FSC-H gating.

4 Statistical analysis

All statistical analyses were performed using Statistical Product and Service Solutions (SPSS) (version 25.0, IBM, Armonk, NY, USA) software. The Mann-Whitney U test was used to analyze data between patients with MM and HIs for two independent samples, whereas the Wilcoxon signed-rank test was used to compare TOX expression in T cell subsets between BM and PB for two related samples.
