# Supplementary material for: Increased TOX expression associates with exhausted T cells in patients with multiple myeloma
Source: Exp Hematol Oncol. 2022 Mar 4;11:12. doi: 10.1186/s40164-022-00267-0 (PMC8895562; doi:10.1186/s40164-022-00267-0)
Supplement: Supplementary file 3 — Additional file 3: Table S1. Clinical information of MM patients used in the study. [file 40164_2022_267_MOESM3_ESM.docx]

**Table S1 Clinical information of MM patients used in the study**

| **Case No.** | **Sex** | **Age** | **MC** | **R-ISS** | **Diagnosis time** | **BM proplasmacyte cell (%)** | **mSMART3.0** | **Therapy** | **Response** |
| --- | --- | --- | --- | --- | --- | --- | --- | --- | --- |
| P1 | F | 67 | IgG | II | 2020.12.03 | 51 | HR | VCD | Loss (2020.12.25) |
| P2 | F | 58 | IgG | II | 2021.01.15 | 50 | SR | IRD | PR (2021.09.27) |
| P3 | M | 66 | Light chain | I | 2021.04.12 | >10% (pathology) | SR | VRD | Loss (2021.11.20) |
| P4 | M | 56 | IgG | II | 2021.05.31 | 28 | SR | VCD | MR (2021.10.17) |
| P5 | M | 62 | IgA | I | 2021.06.02 | 38.5 | SR | IRD | MR (2021.08.03) |
| P6 | M | 44 | IgG | II | 2021.06.28 | 39 | SR | VCD | Loss (2021.11.20) |
| P7 | F | 62 | IgG | III | 2021.07.02 | 55 | HR | VCD | PR (2021.10.17) |
| P8 | F | 36 | Light chain | II | 2021.06.24 | >10% (pathology) | SR | VRD | CR (2021.10.17) |
| P9 | M | 72 | Light chain | III | 2021.07.19 | 30 | HR | ITD | CR (2021.10.17) |
| P10 | F | 62 | IgG | III | 2021.07.22 | 38 | HR | VCD | MR (2021.10.17) |
| P11 | F | 63 | IgG | II | 2021.07.31 | 44.8 | HR | PAD,VD | SD (2021.10.11) |
| P12 | M | 73 | IgA | III | 2021.08.04 | 60 | HR | VCD | Loss (2021.11.20) |
| P13 | M | 60 | IgA | II | 2021.08.13 | >10% (pathology) | HR | VTD | CR (2021.12.10) |
| P14 | M | 58 | IgA | III | 2021.08.18 | >10% (pathology) | HR | VCD | PR (2021.10.17) |
| P15 | M | 64 | IgG | III | 2021.08.22 | 26.5 | HR | VD | SD (2021.10.11) |
| P16 | M | 41 | IgD | III | 2021.09.07 | 45 | HR | VCD | MR (2021.10.18) |

Notes: MM: multiple myeloma; MC: monoclonal component; R-ISS: Revised International Staging System; mSMART 3.0: Mayo Stratification for Myeloma and Risk-Adapted Therapy (https://www.msmart.org/mm-treatment-guidelines); SR: Standard-Risk; HR: High-Risk; VCD: bortezomib cyclophosphamide and dexamethasone; IRD: ixazomib, lenalidomide, and dexamethasone; VRD: bortezomib, lenalidomide, and dexamethasone; ITD: ixazomib, thalidomide, and dexamethasone; PAD: bortezomib, doxorubicin, and dexamethasone; VTD: bortezomib, thalidomide, and dexamethasone; VD: bortezomib and dexamethasone; CR: complete response; PR: partial response; MR: minimal response; SD: stable disease.
